# The efficacy of an allosteric modulator of the alpha 7 nicotinic acetylcholine receptor in a murine model of stroke

**DOI:** 10.3389/fnins.2025.1525975

**Published:** 2025-02-12

**Authors:** Katherine Hernandez, Nathan Jones, Sterling B. Ortega

**Affiliations:** Department of Microbiology, Immunology, and Genetics, University of North Texas Health Science Center, Fort Worth, TX, United States

**Keywords:** PNU-120596, CD4 T-cells, stroke, TMCAO, neuroinflammation

## Abstract

**Introduction:**

Ischemic strokes contribute significantly to cardiovascular-related deaths in the U.S., with current interventions limited to thrombolytic agents. However, these agents present challenges such as a limited therapeutic window, incomplete reperfusion rates, risk of transformation, reperfusion-induced inflammation, and a lack of promoting neuroprotection. We investigated an additional strategy in which prior studies indicated a neuroprotective role. Using a murine transient middle cerebral artery occlusion (tMCAO) model, we sought to evaluate the neurotherapeutic efficacy of a positive allosteric modulator of the alpha7 nicotinic acetylcholine receptor (α7-nAChR), PNU-120596 (PNU), specifically examining whether PNU would modulate stroke-induced neurological dysfunction and neuropathology, with modulation of neuroinflammation as a possible mechanism.

**Methods:**

Young male C57BL/6J mice received a subcutaneous injection of 20mg/kg of vehicle (DMSO) or PNU-120596 immediately after reperfusion, and infarct area and Bederson score were analyzed 24 hours post-stroke. In the 72-hour post-stroke study, the animals were injected with 20mg/kg of PNU or vehicle subcutaneously immediately after reperfusion, followed by two additional doses of 10mg/kg of PNU or vehicle at 24 and 48 hours post-tMCAO. Seventy-two hours later, behavior function and infarct area were assessed.

**Results:**

In contrast to previous rat studies that demonstrated improvements in clinical outcomes, a single administration of PNU following stroke induction led to a reduction in acute neuropathology but did not produce a significant improvement in motor outcomes. Prolonged treatment showed no significant changes in acute neuropathology or sensorimotor function. Additionally, an assessment of neuroinflammation revealed no changes in CD4 T-cell cellularity or phenotype.

**Discussion:**

These findings, alongside prior studies, suggest that the therapeutic efficacy of PNU may be contingent upon the timing of administration, dosage, and pharmacokinetics.

## 1 Introduction

In the United States, one in six deaths attributed to cardiovascular disease is caused by a stroke, with more than 795,000 people experiencing a stroke each year (Catanese et al., 2017). Currently, there is only one FDA-approved pharmaceutical thrombolytic option for stroke called alteplase (Catanese et al., 2017). Alteplase is a recombinant tissue plasminogen activator (tPA) with a narrow therapeutic window of 0–4.5 h after stroke onset and is only designed to target the clot. However, even when complete reperfusion is attained, approximately only 50% of patients experience neurological recovery (Dhillon, 2012; Neumann-Haefelin et al., 2004). Therefore, exploring additional therapeutic options is crucial, especially since there are no FDA-approved therapies that specifically promote post-stroke recovery.

The pathophysiology of stroke is characterized by neuronal loss due to central nervous system (CNS) ischemia and the subsequent induction of an inflammatory response, which has been shown to induce secondary neuropathology (Wang et al., 2007). Various immune cells, including CD4 T-cells, are activated after an ischemic stroke. In a study examining the role of CD4 T-cells in stroke, Zhang et al. (2018) induced a transient middle cerebral artery occlusion (tMCAO) in major histocompatibility complex (MHC) II knockout mice and observed a smaller infarct volume as compared to the wildtype control, supporting the idea that the presence of CD4 T-cells is detrimental in stroke. Similar results were found in a study where an antibody depletion protocol was used to remove CD4 T-cells. In this study, CD4 T-cell depleted tMCAO mice demonstrated improved neurological scores and increased contralateral movement, a motor deficit commonly observed in the tMCAO model, compared to the control-treated mice (Harris et al., 2020). However, there are several CD4 T-cell subtypes. In a study evaluating T helper (Th) 1, Th2, and Th17 CD4 T-cells in acute ischemic stroke patients, an increase in both Th1 and Th17 cells was observed, with no significant difference in the Th2 subset compared to controls. Furthermore, when comparing the National Institute of Health Stroke Score Scale (NIHSS), a tool used to assess the severity of stroke, the researchers found that that patients with higher levels of Th1 and Th17 cells exhibited worse NIHSS scores (Yu et al., 2022). This correlation suggests that CD4 T-cells play a significant role in stroke pathology and recovery, making them a critical focus of interest.

A novel drug PNU-120596 (PNU), a positive allosteric modulator (PAM) of the alpha7 nicotinic acetylcholine receptor (α7-nAChR), has demonstrated promising results in reducing infarct volumes and improving motor function, primarily in a rat stroke model (Gaidhani and Uteshev, 2018). PNU is classified as a type II PAM, meaning it does not bind to the orthosteric site where agonists and antagonists compete. Instead, it binds to the transmembrane domain and inhibits receptor desensitization, making it a compelling therapeutic agent (Sun et al., 2013). The α7-nAChR is expressed in various central nervous system cells, and additional studies have shown that α7-nAChR is also expressed in immune cells, with increased expression observed in activated inflammatory CD4 T-cells (Nizri et al., 2009).

However, the target cell and mechanism of action of PNU remain to be fully determined. In addition to its direct neuroprotective role, PNU’s ability to modulate stroke-induced neuroinflammation is unknown. Elucidating this is crucial, as various stroke models have demonstrated the secondary neuropathological effect of stroke-induced CD4 T-cells. Supported by the current literature, we hypothesize that PNU will be effective in a mouse stroke model, and PNU employs an additional mechanism that regulates stroke-induced neuroinflammatory CD4 T-cells. In this study, we sought to investigate the therapeutic potential and mechanism of action of an α7-nAChR PAM, PNU-120596, using a mouse tMCAO model, specifically examining whether PNU would modulate stroke-induced neurological dysfunction and neuropathology, with modulation of neuroinflammation as a possible mechanism.

## 2 Materials and methods

### 2.1 Mice

Young male C57BL/6J mice (9–12 weeks old) were purchased from Jackson Laboratory (Bar Harbor, ME). All animals were housed in climate-controlled, pathogen-free facilities under veterinary supervision. They were under a 12 h light and dark cycle with access to food and water *ad libitum*. All experiments were conducted using the ARRIVE guidelines and were approved by the University of North Texas Health Science Center Institutional Animal Care and Use Committee (IACUC 2021-0045).

### 2.2 Murine stroke model

All experimental time points were conducted on a minimum of two separate replicates. Similar to our previous report but briefly described here, the transient middle cerebral artery occlusion (tMCAO) procedure was performed by a surgeon who anesthetized the animal using isoflurane (1.8–2%), 70% nitrous oxide, and 30% oxygen (Ortega et al., 2020). After the animal was anesthetized, they were placed on a heating pad to maintain a constant body temperature throughout the procedure. For the surgical process, an incision was made on the carotid bifurcation, the left carotid artery was ligated, and a 0.23 mm in diameter nylon filament (Cat 602334PK10, Doccol Corporation) was inserted into the internal carotid artery and advanced to the middle cerebral artery. The filament remained in place for 60 min before being removed to allow for reperfusion. Cerebral blood flow and baseline reductions were measured using a Laser Doppler (moorVMS-LDF 1, Moor Instruments), with a successful tMCAO defined by an 80% reduction in cerebral blood flow and a 50% baseline recovery following reperfusion. A neurological assessment was also performed for inclusion. Animals that did not display circling behavior were excluded from the study. Post-surgery, animals received local anesthetics, including bupivacaine, to support recovery and were closely monitored for any signs of distress. If there were any signs of distress, the animals were removed from the study.

### 2.3 α7-nAChR positive allosteric modulator

Upon confirmation of successful occlusion and reperfusion, the animals received the vehicle (DMSO) or the α7-nAChR PAM PNU-120596 (reconstituted in 100% DMSO). The injections were administered randomly, and the individual administering the injections was blinded to the treatment groups. Based on body weight, each animal received up to 100 μL per injection throughout the experimental course. There were no signs of toxicity observed, such as skin lesions, hair loss, or any other adverse effects due to DMSO administration. In the 24 h post-stroke study, the animal was administered 20 mg/kg of vehicle or PNU-120596 subcutaneously immediately after reperfusion. Twenty-four hours later, behavior function and infarct percentage were assessed. In the 72 h post-stroke study, the animals were injected with 20 mg/kg of PNU or vehicle subcutaneously immediately after reperfusion, followed by two additional doses of 10 mg/kg of PNU or vehicle at 24 and 48 h post-tMCAO (referred to as 20-D0 × 10-D1 × 10-D2). Seventy-two hours later, behavior function and infarct percentage were assessed.

### 2.4 Behavioral Assessments

To study stroke-induced unilateral brain damage, we used the Adhesive Removal Test. Briefly, a small piece of adhesive tape is placed on each animal’s forepaw, and the time in which the animal contacts the adhesive tape and removes it is measured, with the maximum time per trial being 120 s (Bouet et al., 2009).

The Corner Test was used to assess sensorimotor dysfunction by positioning two acrylic boards together at a 30° angle. The mice were then placed in the center of the apparatus, and the direction of their turn (left or right) was recorded. Each animal underwent five trials per day with a 30 s rest in between trials. The corner turning (CT) score was analyzed as CT score = [(Right turn)/(Right turn + Left turn)] × 100 (Lekic et al., 2012).

Neurological deficits were assessed using the Bederson score, with the following scoring criteria: 0 = no deficits, 1 = forelimb flexion, 2 = decreased resistance to lateral push, 3 = circling.

### 2.5 Infarct percentage assessment

Using the 2,3,5-Triphenyltetrazolium Chloride Stain (TTC) assay (Cat A10870.30, Thermofisher, United States), brain tissue was collected, and the olfactory region was removed to create approximately 1 mm sections using a brain matrix. The brain slices were placed in a petri dish with 2% TTC for 7 min. After 7 min, the slices were flipped and incubated for another 8 min in the TTC solution. Following the incubation, the samples were transferred to a glass vial containing 4% paraformaldehyde (Cat. 50980487, Fisher Scientific, United States) in order to stop the TTC reaction. The samples were subsequently imaged using an Epson Perfection V600 Photo Scanner, and the infarct and contralateral brain section areas were measured using ImageJ version 1.53 software. To account for edema the following equation was used to calculate the infarct percentage of each slice: $\left(I\,A=\frac{\left(T\,C\,A-N\,I\,I\,A\right)}{T\,C\,A}\times 100\right)$ (IA = infarct area percentage; TCA = total contralateral brain section area; NIIA = non-infarcted ipsilateral brain section area). The infarct percentage of each animal was the mean of all the slices (3-5 slices) (Gaidhani and Uteshev, 2018).

### 2.6 Sample preparation for functional assessment

Spleens were collected and filtered through a 70 μm nylon filter, followed by washing with fresh cell culture media containing 10% Fetal Bovine Serum (FBS) (Cat. 100-106-50, Gemini Bio), 1.25% HEPES Buffer (Cat. 15630080, Gibco), 1% non-essential amino acids (Cat. 11140050, Gibco), 1% of 100x Penicillin-Streptomycin (Cat. 15140122, Gibco), 1% of 200 mM L-glutamine (Cat. 25030081 Gibco), and 0.0002% 2-Mercaptoethanol (Cat. M3148-25ML, Millipore Sigma) in 1X Roswell Park Memorial Institute Medium (RPMI) (Cat. MT10040CV, Corning). Single-cell suspensions were generated by overlaying the suspension over Lympholyte-M (Cat. CL5035, Cedarlane). Buffy coat layer was carefully collected and the suspension was washed twice with fresh culture media. Cervical lymph nodes were collected and processed using a 70 μm nylon filter and washed with fresh media. All cell counts were obtained using 4% trypan blue (Cat. 15250061, Gibco) and a Cytosmart cell counter (Corning).

### 2.7 Intracellular cytokine staining for functional assessment

The single-cell suspensions (cervical lymph node or spleen) were incubated with the Cell Stimulation Cocktail (Cat. TNB-4975-UL100, Tonbo) for 6 h, as per the manufacturer’s instructions. Next, the cells were washed with FACS buffer [1X Dulbecco’s Phosphate Buffered Saline (PBS) (Cat. 21-0310CV, Corning), 1% of Bovine Serum Albumin (BSA) (Cat. BP1600-100, Fisher Scientific), 0.1% of sodium azide (Cat. S2002, Millipore Sigma)]. To inhibit non-specific staining, the cells were incubated with Fc Receptor Blocker (Cat. 130-092-575, Miltenyi Biotec) for 5 min at room temperature. Extracellular staining was performed by incubating cells with anti-mouse CD45 Brilliant Violet 650 (Cat. 103151, Biolegend), anti-mouse CD3e Brilliant Violet 711 (Cat. 100348, Biolegend), and anti-mouse CD4 redFluor 710 (Cat. 80-0042-U100, Tonbo) for 30 min at 4°C in the dark. Cells were then washed with FACS buffer, and permeabilization was performed using the Transcription Factor fixation/permeabilization buffer (Cat. TNB-0607-KIT, Tonbo). Cells were then washed with the Permeabilization Buffer from the kit (Cat. TNB-0607-KIT, Tonbo) and incubated with 2% rat serum (Cat. 1355, Stem cell technologies) for 15 min at room temperature. After incubation, the following antibodies were added: anti-mouse interferon gamma VioletFluor 450 (Cat. 75-7311-U100, Tonbo), anti-mouse Foxp3 PE-Cyanine 7 (Cat. 60-5773-U025, Tonbo), anti-mouse IL-17A FITC (Cat. 506907, BioLegend), and anti-mouse IL-4 PE (Cat. 50-7041-U025, Tonbo). The samples were then incubated at room temperature for 30 min in the dark. After incubation, the cells were washed with flow cytometry permeabilization buffer. Finally, 1% paraformaldehyde (Cat. 50980487, Fisher Scientific) was added to fix the samples. The samples were immediately processed using the Aurora flow cytometer (Cytek), and data was analyzed using FlowJo software (BD Bioscience).

### 2.8 Statistical analysis

All statistical analyses were performed using GraphPad Prism v10 and all experiments were conducted by a blinded individual. The sample size was established with an alpha of 0.05, a power of 0.85, and an effect size of two, similar to other studies (Gaidhani and Uteshev, 2018; Sun et al., 2013). The sample size was analyzed using G*Power 3.1 software. The behavioral data was analyzed using a two-way mixed measures ANOVA followed by Tukey’s multiple comparisons test to compare multiple groups. In addition, the Mann-Whitney U test was used to assess neurological scores, infarct percentage, and flow cytometry data.

## 3 Results

### 3.1 Immediate administration of PNU in a murine model of stroke leads to decreased infarct percentage without significant change in outcomes

**FIGURE 1:**
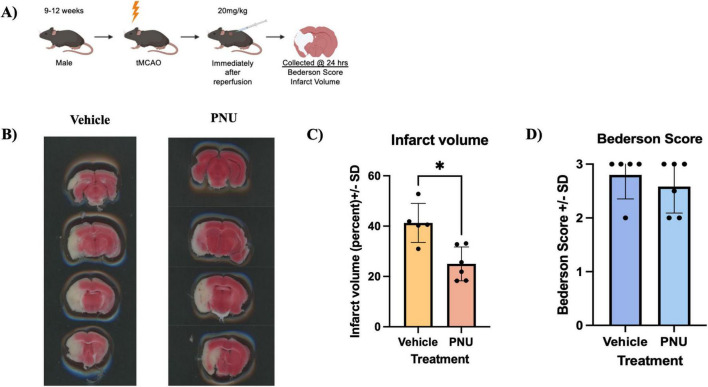
A 20 mg/kg dosage of PNU-120596 (PNU) given immediately after transient cerebral artery occlusion (tMCAO) reduces infarct percentage in the acute phase of stroke. **(A)** A schematic of the experimental design is presented. **(B)** 2,3,5-Triphenyltetrazolium Chloride (TTC) images from vehicle-treated and PNU-treated animals are shown. **(C)** Infarct percentage was quantified 24 h after reperfusion, showing a significant reduction in infarct percentage in the PNU-treated group compared to the vehicle-treated group. **(D)** Behavioral analysis showed no significant changes in their Bederson score in the PNU-treated group compared to the vehicle-treated group. The data represents two independent experiments, and the mean ± standard deviation (SD) is shown. The non-parametric data was analyzed using the Mann-Whitney U test; vehicle *n* = 5 mice, PNU *n* = 6 mice, *p < 0.05, **p < 0.01.

Clinical trials of neurotherapeutics focused on stroke recovery thus far have failed to demonstrate a higher treatment efficacy as compared to standard rehabilitation regimens (Rogalewski and Schäbitz, 2021). Previous studies have shown that administration of PNU following tMCAO induction led to a profound reduction in infarct volume and improved functional outcomes in rats (Sun et al., 2013). Thus, we performed an experimental setup using a murine stroke model whereby 9–12 weeks-old mice underwent 60 min tMCAO procedures. The mice were separated into two cohorts, each with similar neurological deficits and then treated with subcutaneous administration of 20 mg/kg of vehicle or PNU (Figure 1A). To measure changes in neuropathology, brain samples were collected 24 h post-treatment and infarct volumes were measured using the 2,3,5-triphenyltetrazolium (TTC) assay (Figure 1B). A single dose of PNU significantly reduced infarct percentage compared to the vehicle-treated group (Figure 1C). However, neurological impairment, assessed immediately prior to tissue harvest using the Bederson score, did not differ significantly between the PNU and vehicle-treated groups (Figure 1D). Taken together, while a single administration of PNU did reduce stroke neuropathology, no significant improvement in neurological dysfunction was observed.

### 3.2 Multiple dosages of PNU does not alter infarct percentage or gross motor dysfunction in a murine model of stroke

**FIGURE 2:**
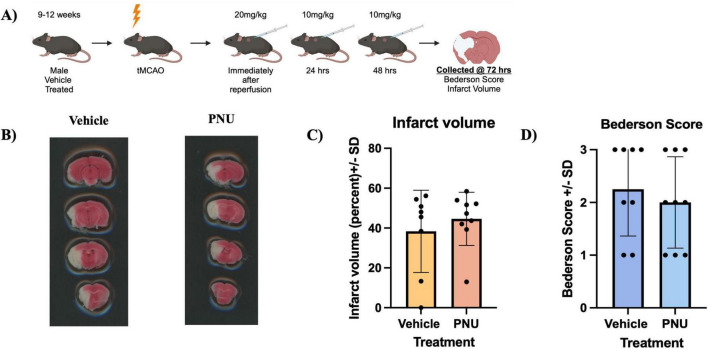
A higher frequency and increased total dose (20-D0 × 10-D1 × 10-D2) of PNU-120596 (PNU) after transient cerebral artery occlusion (tMCAO) does not reduce infarct volume in the acute phase of stroke. **(A)** A schematic illustrating the administration of PNU is presented. **(B)** 2,3,5-Triphenyltetrazolium Chloride (TTC) images from vehicle-treated and PNU-treated animals are shown. **(C)** Infarct percentage at 72 h post-tMCAO were quantified, with no significant difference between the vehicle and PNU-treated groups. **(D)** Behavioral assessment using the Bederson score showed no significant changes in the PNU-treated group compared to the vehicle. The data represents three independent experiments, presented as the mean ± SD. Data was analyzed using the Mann-Whitney U test; vehicle *n* = 8 mice, PNU *n* = 9 mice.

Previous literature has demonstrated that a three dose regimen, which mimics the drip infusion method in a hospital setting, results in a reduction of neuropathology and improved neurological function 72 h after stroke induction in rats (Gaidhani and Uteshev, 2018). Having observed a suboptimal effect with a single dose of PNU (Figure 1), we proceeded to recapitulate the previously successful drip method (Gaidhani and Uteshev, 2018) with 20 mg/kg of PNU at day 0, and 10 mg/kg of PNU at 24 and 48 h post-tMCAO (20-D0 × 10-D1 × 10-D2) (Figure 2A). Interestingly, increasing the frequency and amount of treatment did not reveal a change in neuropathology 72 hours after a stroke induction in mice (Figures 2B, C). Understandably, the evaluation of neurological function did not reveal a change in the Bederson score in mice having undergone PNU treatment using the drip method (Figure 2D).

### 3.3 PNU treatment does not alter sensory dysfunction in a murine model of stroke

**FIGURE 3:**
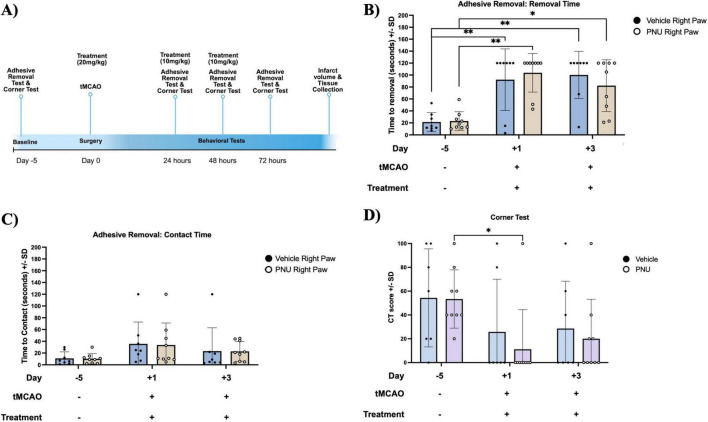
The 20-D0 × 10-D1 × 10-D2 PNU-120596 (PNU) dosage treatment in mice does not result in changes to their asymmetrical deficits. **(A)** A schematic outlining the timeline of the behavioral tests is presented. **(B)** In the Adhesive Removal Test, there is an increase in the time required to remove the adhesive on both day one and day three post-transient cerebral artery occlusion (tMCAO) for both groups as compared to the training time point. Both vehicle- and PNU-treated groups did not differ in time to remove the adhesive. **(C)** In the time to contact the adhesive, no significant difference were observed across all three time points. **(D)** In the Corner Test, the CT score decreased on day one post-tMCAO compared to the baseline in the PNU-treated group. However, no significant difference was observed at day three post-tMCAO when compared to both day one and the baseline. The parametric data represents three independent experiments, presented as the mean ± SD. The data are analyzed using a two-way mixed measures ANOVA followed by Tukey’s multiple comparisons test; vehicle *n* = 7 mice, PNU *n* = 9 mice; *p < 0.05, **p < 0.01.

Having determined that PNU treatment did not alter gross stroke-induced neurological dysfunction in mice, we next wanted to determine if clinical efficacy was limited to fine motor movements using the Adhesive Removal Test. A baseline performance evaluation was performed 5 days prior to stroke induction (Figure 3A). Having completed training and immediately prior to stroke induction, both the PNU-treated and vehicle control groups exhibit similar performance levels (∼21 and 23 s to remove the adhesive, Figure 3B). Similar to our prior experiments, we induced a 60 min tMCAO and treated the mice using the 20-D0 × 10-D1 x 10-D2 dosage regimen. We then assessed somatosensory deficits at 24, 48, and 72 h post-stroke induction. One day post-stroke induction and after at least two PNU treatments, the time to remove the adhesive increased similarly, in both groups (∼90 and 92 s). At 3 days post-stroke, after receiving all PNU treatments, we did not see a difference in time to removal between the PNU and vehicle-treated group (Figure 3B). Similarly, the time to contact the adhesive was increased after stroke (day 1 and 3) but no difference was observed between the vehicle and PNU-treated groups (Figure 3C).

Additionally, we assessed sensorimotor and postural asymmetries using the Corner Test. Both vehicle- and PNU-treated mice exhibited a CT score of 50 at baseline. One day after stroke induction and treatment administration, the CT score decreased to ∼25 and 14, vehicle and PNU treatment, respectively, but there were no significant differences between the groups (Figure 3D). Similarly, 3 days post-stroke/treatment induction, no differences were observed between PNU- and vehicle-treated groups (Figure 3D). In this mouse model of stroke, administration of PNU did not alter sensorimotor recovery.

### 3.4 Post-stroke neuroinflammation is not altered by PNU treatment

**FIGURE 4:**
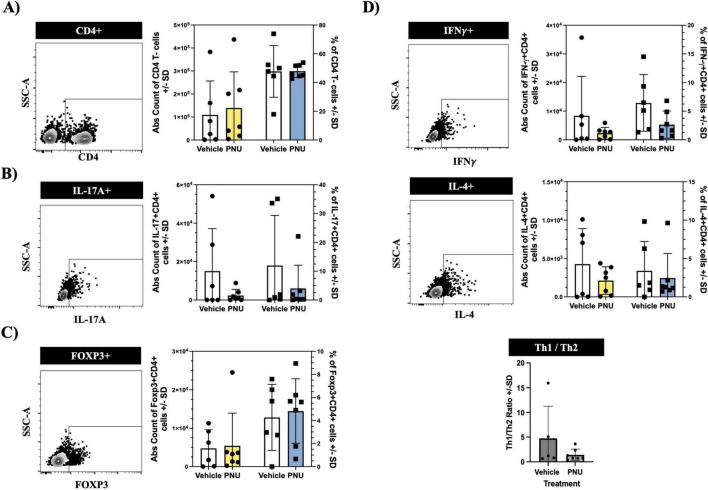
PNU-120596 (PNU) administration does not alter the CD4 T-cell phenotype in the central nervous system (CNS)-relevant immune compartment. **(A)** Representative dot plot showing the CD4 gate on CD3^+^ events isolated from the cervical lymph nodes. In the bar graph, the left two columns indicate absolute counts of CD4 T-cells, while the right two columns indicate the relative percentages. There were no significant differences in the absolute count and percentages of CD4 T-cells between the vehicle and PNU-treated group. **(B)** Representative data showing the IL17^+^ gate on CD4^+^ events. Similarly, no changes were observed in the pathogenic Th17 (CD4^+^IL-17A^+)^ T-cells in either the absolute count or percentages. **(C)** Representative data showing the Foxp3^+^ gate on CD4^+^ events. The absolute count and percentages of regulatory (CD4^+^Foxp3^+^) T-cells remained unchanged. **(D)** Representative data showing the IFN-γ^+^ gate or IL4^+^ gate on CD4^+^ events. The pathogenic Th1 (CD4^+^IFNy^+^) and anti-inflammatory Th2 (CD4^+^IL-4^+^) T-cells showed no significant differences in absolute values and percentages between the two groups. The Th1/Th2 ratio also showed no difference between the vehicle and PNU-treated group. The data represents three independent experiments, shown as the mean ± SD, and was analyzed using the Mann-Whitney U test; vehicle *n* = 6 mice, PNU *n* = 7 mice for all graphs except the Th1/Th2 ratio (vehicle *n* = 5 mice, PNU *n* = 7 mice).

**FIGURE 5:**
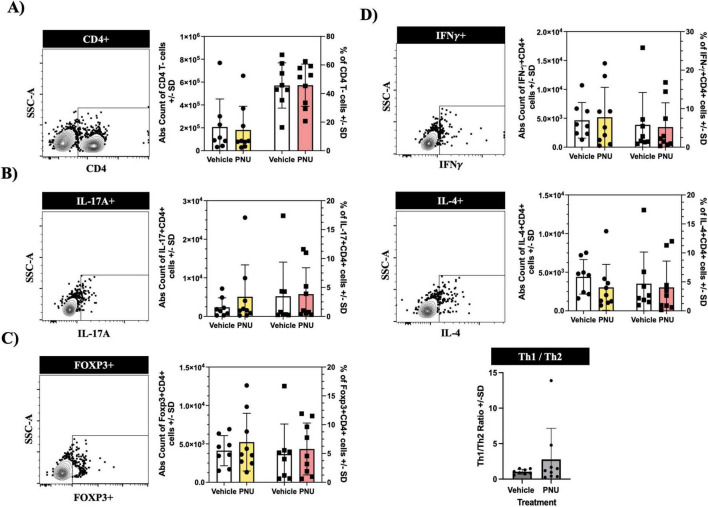
PNU-120596 (PNU) administration does not alter the CD4 T-cell phenotype in the peripheral immune compartment. **(A)** Representative dot plot showing the CD4 gate on CD3^+^ events isolated from the spleen. There were no significant differences in the absolute count and percentages of CD4 T-cells between the vehicle and PNU-treated group. **(B)** No significant changes were observed in the pathogenic Th17 (CD4^+^IL-17A^+^) subsets in both the absolute count and percentage. **(C)** Regulatory T-cells (CD4^+^Foxp3^+^) also exhibited no difference in either their absolute count or percentages. **(D)** The pathogenic Th1 (CD4^+^INF-y^+^) and anti-inflammatory Th2 (CD4^+^IL-4+^+^ T-cells showed no significant difference in their absolute counts and percentages between the two cohorts. The Th1/Th2 ratio also showed no differences between the vehicle and PNU-treated group. The data represents three independent experiments, shown as the mean ± SD, and was analyzed using the Mann-Whitney U test; vehicle *n* = 9 mice, PNU *n* = 9 mice.

Following a stroke, ischemic injury triggers a cascade of events leading to the activation of immune cells (Ortega et al., 2020). These immune cells, from both the innate and adaptive compartments (Ortega et al., 2015), produce pro-inflammatory cytokines and chemokines, which exacerbate neuronal damage and contribute to secondary injury (Khoshnam et al., 2017). Among these inflammatory responses, pro-inflammatory CD4 T-cells have been identified as key neuropathogenic cells (Ma et al., 2021). Similarly, regulatory CD4 T-cells have also been implicated in neuropathology (Kleinschnitz et al., 2013). Thus, we aimed to investigate whether PNU could modulate these pathogenic cells. Having undergone stroke induction and PNU treatment, we harvested the draining lymph nodes of the brain, as well as the spleen, and quantified inflammatory CD4 T-cells using flow cytometry. Using a rigorous gating strategy that removes doublets and isolates T-cells by gating on CD45+CD3+ (Supplementary Figure 1, CD45 and CD3 cellularity data in Supplementary Figures 2, 3), we quantified CD4 T-cells by gating on CD4+ cells (Figure 4A, left dot plot) and extrapolated counts using the hemacytometer counts and percentage of specific cell population. Cellular quantification at day three post-stroke did not reveal changes in absolute counts (Figure 4A, bar graph – left two columns) or percentages (Figure 4A, bar graph – right two columns) of CD4 T-cells in either the draining lymph node of the brain (cervical lymph node, Figure 4A) or the peripheral immune system (spleen, Figure 5A). Next, we evaluated the cellularity of inflammatory CD4 T-cells (Th17). Compared to mice having undergone tMCAO and received vehicle treatment, mice having received PNU post-stroke did not show a change (count or percentage) of inflammatory Th17 (CD4^+^IL17^+^) cells in the draining lymph nodes (Figure 4B) or spleen (Figure 5B). Evaluation of regulatory (CD4^+^Foxp3^+^) T-cells did not reveal a change (count or percentage) in the draining lymph nodes (Figure 4C) or spleens (Figure 5C) or spleens of PNU-treated mice. Finally, evaluation of the inflammatory Th1 (CD4^+^IFN-g^+^) and anti-inflammatory Th2 (CD4^+^IL4^+^) T-cells also did not reveal a change (count or percentage) in PNU-treated post-stroke mice. It has been shown that treatments that modulate neuroinflammation may also change the ratio of Th1 to Th2 cells (Nizri et al., 2009). Evaluation of the Th1 to Th2 ratio in our PNU-treated mice did not reveal a change between the two groups (cervical lymph node, Figure 4D; spleen, Figure 5D). In summary, administering PNU using the 20-D0 × 10-D1 × 10-D1 dosage did not alter the inflammatory immune response in this murine stroke model.

## 4 Discussion

Previous reports have highlighted the targeting of the α7 nicotinic acetylcholine receptor and its role as a neuroreparative agent (Gaidhani and Uteshev, 2018; Nizri et al., 2009; Sun et al., 2013). This study aimed to further evaluate the efficacy of PNU treatment as a promoter of neurorecovery in the context of stroke. The plan was to validate the efficacy by evaluating changes in neurological function and neuropathology, and possible mechanisms by evaluating changes in neuroinflammation.

α7-nAChRs are a subtype of nicotinic receptors widely expressed in CNS and peripheral immune system cells, including microglia (Simone et al., 2005), astrocytes (Han et al., 2014), macrophages (Wang et al., 2003), lymphocytes (Rosa et al., 2005), and endothelial cells (Vieira-Alves et al., 2020). These receptors are ligand-gated ion channels that mediate rapid signal transmission by being the target of the neurotransmitter acetylcholine. In the CNS, they are predominantly expressed in neurons and glia of the cortex and hippocampus, playing crucial roles in cognitive functions such as learning and memory (Letsinger et al., 2022). Targeting CNS cells expressing α7-nAChRs has revealed their crucial role in neurodevelopment (Wu et al., 2015), neuroprotection (Gaidhani and Uteshev, 2018), neurodegenerative diseases (Callahan et al., 2013; Gowayed et al., 2022), and psychiatric disorders (McLean et al., 2012; Targowska-Duda et al., 2021).

Similarly, targeting immune cells expressing α7-nAChRs has revealed their role in neuroinflammation. Activation of cells through α7-nAChRs has been shown to induce an anti-inflammatory milieu by modulating the release of pro-inflammatory cytokines (Nizri et al., 2009). Agonist and PAMs are currently being explored as potential agents of neuroprotection by promoting anti-inflammatory milieu and neuroplasticity in conditions like stroke (Han et al., 2014), multiple sclerosis (Godin et al., 2020), chronic pain (Freitas et al., 2013), and sepsis (Shi et al., 2022).

Some α7-nAChR agonists can penetrate the blood-brain barrier, making them considerable candidates for targeting cognitive dementia and schizophrenia (Pohanka, 2012). For example, RG3487, an α7-nAChR agonist and serotonin receptor antagonist, has shown efficacy in modulating cognitive function in rodents and holds potential for treating Alzheimer’s disease (Wallace et al., 2011). Similarly, the agonist SEN34625/WYE-103914 has shown potential in treating cognitive disorders (Haydar et al., 2009), while the ABT-107 agonist demonstrated neuroprotective effects by protecting rat cortical cultures from glutamate-induced toxicity (Malysz et al., 2010). In stroke models, several α7-nAChR agonists and antagonists have been described. Using a permanent middle cerebral artery occlusion murine model, PHA 568487-(agonist) treated mice were found to perform better on neurofunctional tests, had decreased infarct volumes, and less neuronal cell death, which was reversed in mice treated with the antagonist methyllycaconitine (MLA) (Han et al., 2014).

Ischemic injury in the CNS leads to the development of neuroinflammation, a physiological process that is initially beneficial by removing injured/dead cells and initiating tissue regeneration. However, excessive and long-term inflammation after a stroke has been shown to lead to secondary neuropathology (Wang et al., 2007). Thus, targeting and suppressing this excessive neuroinflammation is paramount to promoting neurological recovery after a stroke. α7-nAChRs have been shown to be expressed on microglia (CNS endogenous innate cell) and macrophages (peripheral innate cell). In fact, treatment by exogenous nicotine or the neurotransmitter acetylcholine (ACh) has been shown to inhibit the production of pro-inflammatory cytokines in macrophages, a phenomenon absent in cells obtained from α7 knockout mice, termed the cholinergic anti-inflammatory pathway (Jonge and Ulloa, 2007; Wang et al., 2003). A similar observation was observed when microglia and astrocytes were treated with ACh (Egea et al., 2015; Patel et al., 2017; Shytle et al., 2004). This immune inhibition is believed to occur through two pathways: Jak2/STAT3 and PI3K/Akt (Bencherif et al., 2011; Jonge et al., 2005). The Jak2/STAT3 metabotropic signaling results in the inhibition of the nuclear factor (NF)-kB, which is involved in the expression of several inflammatory cytokines including, IL1β, IL6, and TNF-α (Egea et al., 2015). Similarly, the PI3K signaling leads to Nrf2 inhibiting NF-kB (Egea et al., 2015). With a similar end result but mediated by PLC signaling, activation of α7-nAChRs in microglia and astrocytes results in an increase in intracellular calcium stores, thereby inhibiting phosphorylation of JNK, p38, and P44/42, kinases involved in neuroinflammation (Lee et al., 2000; You et al., 2018; Youssef et al., 2019).

In our study, a single dose of PNU immediately after reperfusion decreased the infarct percentage at 24 h post-tMCAO. However, when the young mice were treated with the method that mimics the drip infusion given to stroke patients, the infarct percentage did not decrease compared to the vehicle 3 days post-tMCAO. There was no difference in neurological dysfunction between the vehicle and PNU in both treatment regimens. The lack of significant differences in infarct percentage between groups in the multi-dose treatment regimen may be due to the specific dosage and method of administration used, as well as the time of assessment. This supports the idea that PNU’s neuroprotective efficacy may be time-dependent, and its effects are beneficial when administered early, emphasizing the importance of timing in the therapeutic intervention of stroke. It should also be noted that a sham group was not included in testing the efficacy of PNU, as previous studies have shown no significant differences in immune system responses under sham conditions (Morales et al., 2021). Additionally, the differences in observations may be attributed to the possibility that the drug has lost its biological activity; however, this seems unlikely, as experiments demonstrating protection were conducted alongside those in which protection was not observed. In the literature, PNU has demonstrated efficacy in a rat model of stroke (Gaidhani and Uteshev, 2018; Sun et al., 2013), improved cognition in a schizophrenia model (McLean et al., 2012), and shown anti-depressant-like properties (Targowska-Duda et al., 2021). It has also improved motor function in Parkinson’s (Gowayed et al., 2022) and showed better outcomes in an Alzheimer’s disease model (Callahan et al., 2013). However, as we transitioned from a rat to a mouse model, we found that a higher dose of PNU is required to observe efficacy during the first 24 h of a stroke. In addition, it is possible that the observed differences may be due to differences in drug metabolism between species, as previous work has shown efficacy with lower dosages in rats (Gaidhani and Uteshev, 2018).

To account for this, we selected a 20 mg/kg dosage as an intermediate between the 11.5 mg/kg used by Gaidhani and Uteshev (2018) and the 30 mg/kg used by Kalappa et al. (2013) in rat stroke models, both of which administered PNU subcutaneously. Like Gaidhani and Uteshev (2018), we utilized a three-dose injection regimen. However, rather than waiting 6.5 h post-tMCAO, we administered the first dose immediately after reperfusion, followed by additional doses at 24 and 48 h (Gaidhani and Uteshev, 2018). The subsequent doses were half the initial dose [10 mg/kg in our study compared to 5.5 mg/kg in Gaidhani and Uteshev’s (2018) study], following a similar dosing strategy (Gaidhani and Uteshev, 2018). On the other hand, Sun et al. (2013), performed a similar experimental setup but was focused on intravenous injection at a 1 mg/kg dosage.

After establishing that PNU did not alter the general neurological function when using the Bederson score, we wanted to evaluate asymmetrical and sensorimotor coordination. In the adhesive removal test (which tests sensorimotor function), the vehicle and PNU-treated groups showed increased removal times on days 1 and 3 post-tMCAO compared to the training day, with no differences between treatment groups. In the time to contact, no differences were observed between each treatment group. This outcome corresponds with the lack of significant differences in infarct volume between groups in this multi-dose treatment regimen, suggesting that PNU was ineffective at reducing stroke pathology in the multi-dose treatment regimen. In contrast, PHA 568487, a selective α7-nAChR agonist, has been tested in a pMCAO model and has been shown to decrease infarct volume and alleviate the asymmetrical motor deficits in the adhesive removal and corner test during the first 7 days post-pMCAO (Han et al., 2014). This agonist has also improved long-term neurobehavioral deficits in an intracerebral hemorrhage model (Krafft et al., 2017). This further supports the idea that targeting the α7-nAChR is beneficial in stroke therapy.

CD4 T-cells are recruited 24 h after a stroke, but peak infiltration occurs 3–4 days after the stroke (Stevens et al., 2002). While there are a variety of CD4 T-cell subsets, the Th1 subset has shown promotion of stroke neuropathology. Gu et al. (2012) compared Th1, Th2, and a Treg knockout mice and saw a more significant decrease in infarct volume in the Th1 knockout and a lower neurological score compared to the other groups, supporting the hypothesis that the Th1 subset promotes stroke neuropathology. The α7-nAChR is found on CD4 T-cells, and the literature shows that cholinergic signaling can attenuate inflammatory CD4 T-cells, and the receptor expression exponentially increases after the CD4 T-cells become activated (Nizri et al., 2009). In an animal model of multiple sclerosis that was treated with nicotine, an α7-nAChR agonist, the authors saw a decrease in Th1 transcription factor and Th1 cytokines (IFN-y, TNF-a), an increase in Th2 transcription factor GATA-3, a reduction in IL-10, an increase in IL-4, and a decrease in IL-17. Their work demonstrated that α7-nAChR agonist can modulate CD4 T-cells (Nizri et al., 2009). However, our data demonstrated no changes in the inflammatory Th1 (CD4+IFNY+) and Th17 (CD4+IL-17A+), the anti-inflammatory Th2 (CD4+IL-4+), and the T regulatory (CD4+FOXP3+) CD4 T-cell phenotype in the PNU-treated group compared to the vehicle-treated group in both the spleen and cervical lymph nodes. There was also no difference in the Th1 to Th2 ratio between the groups. This may reflect a lack of protection at 3 days post-tMCAO, supporting the time-dependent efficacy of PNU-120596 in the mouse stroke model.

Future research should explore three main areas. First, investigating different dosage regimens and timing is paramount. Especially since type II PAMs could lead to unregulated receptor activation, disrupting calcium homeostasis and causing cell death - particularly during a stroke as choline levels significantly increase (Williams et al., 2012). In our study, PNU administration immediately followed stroke induction, and although this may have an effect on the neurological aspect of stroke, stroke-induced inflammation may not have reached its apex; thus, future research should evaluate delayed PNU administration. Additionally, future research should test varying dosage levels. The pharmacology kinetics in mice may differ significantly, and dosages that were effective in previous rat studies may not be efficacious in mice. For the same reason, future investigations should also consider extending treatment duration.

Second, future studies should look into combination therapies. It may be that PNU might work synergistically with other therapies currently incorporated into the standard of care, including anti-inflammatory drugs, antioxidants, thrombolytic agents, or other alpha 7 partial agonists.

Finally, future studies should evaluate long-term cognitive and sensory outcomes. Our study was limited to outcomes during the acute phases of stroke. Future studies should extend the study to months and years and expand the scope to include spatial learning, memory, and behavior—outcomes significantly affected after a stroke.

## 5 Conclusion

In conclusion, a 20 mg/kg dose of PNU provided initial protection 24 h post-tMCAO. However, the protection is lost at 72 h post-tMCAO and no differences were seen in CD4 T-cells during this time frame. This suggests that the therapeutic window for PNU may be limited in a murine model, which is crucial for optimizing treatment strategies.

## Data Availability

The original contributions presented in this study are included in this article/Supplementary material, further inquiries can be directed to the corresponding author.
