# Supplementary material for: The efficacy of an allosteric modulator of the alpha 7 nicotinic acetylcholine receptor in a murine model of stroke
Source: Front Neurosci. 2025 Feb 12;19:1525975. doi: 10.3389/fnins.2025.1525975 (PMC11860958; doi:10.3389/fnins.2025.1525975)
Supplement: Supplementary file 1 [file Data_Sheet_1.pdf]

Supplementary Material

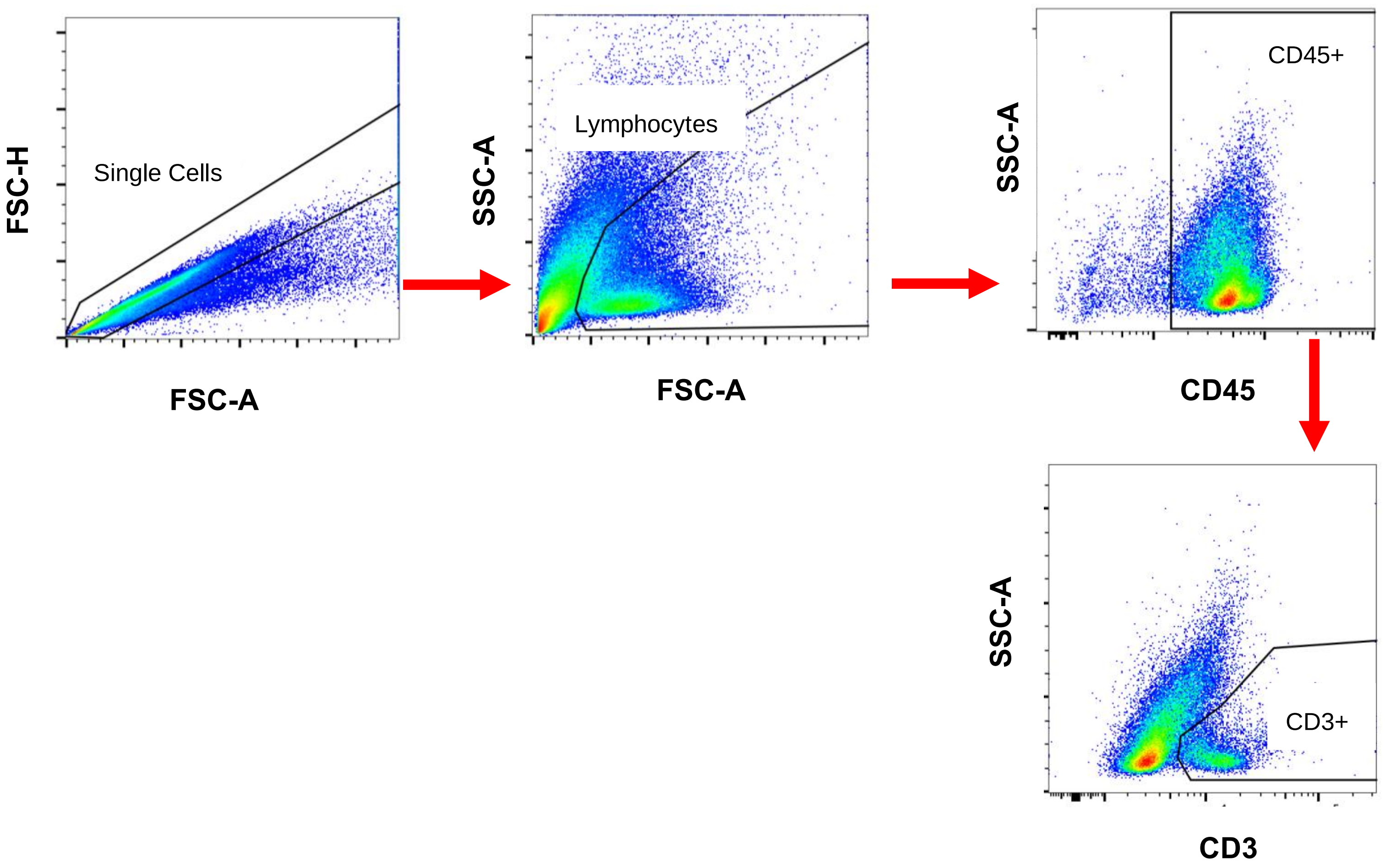

Supplementary Figure 1. A schematic of the gating strategy is shown. Single cells were gated first, followed by the lymphocytes, CD45+ cells, and finally, the CD3+ population. Further gating is shown in the main figures.

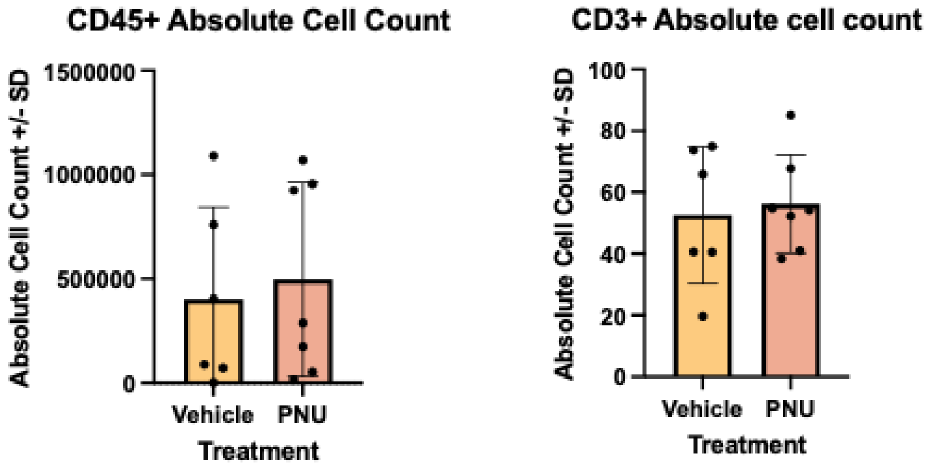

Supplementary Figure 2. Absolute cell count of CD45+ cells (left graph) and CD3+ cells (right graph) in the cervical lymph nodes. The data represents three independent experiments, shown as the mean ± SD, and was analyzed using the Mann-Whitney U test; vehicle n = 6 mice, PNU n = 7 mice

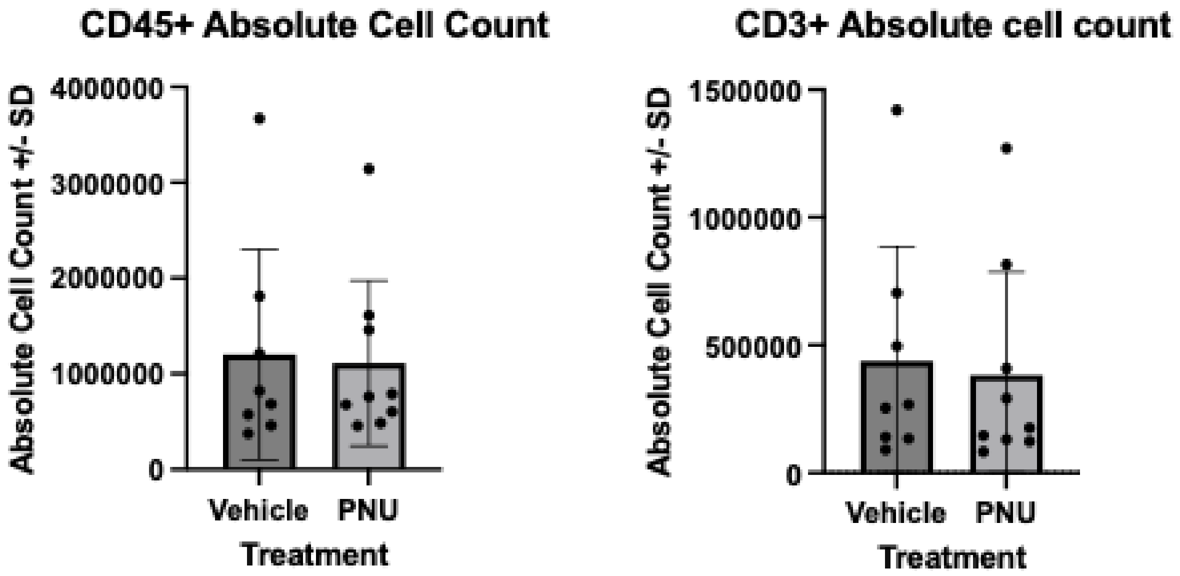

Supplementary Figure 3. Absolute cell count of CD45+ cells (left graph) and CD3+ cells (right graph) in the spleen. The data represents three independent experiments, shown as the mean ± SD, and was analyzed using the Mann-Whitney U test; vehicle n = 9 mice, PNU n = 9 mice.
